# Explorative Data Analysis Methods: Application to Laser-Induced Breakdown Spectroscopy Field Data Measured on the Island of Vulcano, Italy

**DOI:** 10.3390/s23136208

**Published:** 2023-07-07

**Authors:** Kristin Rammelkamp, Susanne Schröder, Alessandro Pisello, Gianluigi Ortenzi, Frank Sohl, Vikram Unnithan

**Affiliations:** 1 Deutsches Zentrum für Luft- und Raumfahrt e.V. (DLR), Institut für Optische Sensorsysteme, 12489 Berlin, Germany; 2 Department of Physics and Geology, University of Perugia, 06123 Perugia, Italy; 3 Deutsches Zentrum für Luft- und Raumfahrt e.V. (DLR), Institut für Planetenforschung, 12489 Berlin, Germany; 4 Department of Physics and Earth Sciences, Constructor University, 28759 Bremen, Germany

**Keywords:** laser-induced breakdown spectroscopy (LIBS), data exploration, field work, planetary analogue site, Aeolian island Vulcano, elemental analysis, geochemical

## Abstract

One of the strengths of laser-induced breakdown spectroscopy (LIBS) is that a large amount of data can be measured relatively easily in a short time, which makes LIBS interesting in many areas, from geomaterial analysis with portable handheld instruments to applications for the exploration of planetary surfaces. Statistical methods, therefore, play an important role in analyzing the data to detect not only individual compositions but also trends and correlations. In this study, we apply two approaches to explore the LIBS data of geomaterials measured with a handheld device at different locations on the Aeolian island of Vulcano, Italy. First, we use the established method, principal component analysis (PCA), and second we adopt the principle of the interesting features finder (IFF), which was recently proposed for the analysis of LIBS imaging data. With this method it is possible to identify spectra that contain emission lines of minor and trace elements that often remain undetected with variance-based methods, such as PCA. We could not detect any spectra with IFF that were not detected with PCA when applying both methods to our LIBS field data. The reason for this may be the nature of our field data, which are subject to more experimental changes than data measured in laboratory settings, such as LIBS imaging data, for which the IFF was introduced first. In conclusion, however, we found that the two approaches complement each other well, making the exploration of the data more intuitive, straightforward, and efficient.

## 1. Introduction

Laser-induced breakdown spectroscopy (LIBS) is a multi-elemental analysis technique that can provide both qualitative and quantitative information about elemental abundances. With a pulsed laser, sample material is ablated, which evolves into a plasma and whose light emissions are collected and analyzed by a spectrometer [1,2,3]. As a result, spectra with typically tens to hundreds of emission lines of different elements being part of the sample matrix are obtained. The LIBS technique meets several criteria, which are important for investigating geological samples in the field; it is fast, samples can be measured in their original environments and instrument designs can be compact even in handheld configuration [4,5,6]. However, the technique also comes with some challenges, as the LIBS spectra are strongly influenced by experimental conditions and so called matrix effects resulting in an often limited repeatability of measurements. Therefore, calibration models which are trained to predict elemental abundances from LIBS data usually have larger uncertainties compared to what can be achieved with high-performing laboratory techniques. Precise and accurate calibration for elemental abundances is particularly challenging when samples in the field are measured where experimental conditions, such as the sample matrix, are usually varying. Thus, besides quantitative calibration models, the use of statistical data exploration methods is common in the LIBS community [7,8,9,10]. Although no absolute values for concentrations are obtained, it enables the identification of similar types of targets (e.g., mineral classification) and provides information about relative elemental enrichments within the analyzed dataset (geochemical trends). Examples include matrix decomposition techniques, such as independent component analysis (ICA) [11], non-negative matrix factorization (NMF) [12], and principal component analysis (PCA) [13,14]. They are often used in conjunction with clustering techniques, like k-means clustering or hierarchical clustering [15,16,17]. Such statistical methods are also interesting to analyze the large datasets which can be generated rapidly and, in a simple way, with LIBS instruments compared to other measurement methods. For example, a measurement with a handheld LIBS instrument takes only a few seconds. LIBS instruments are also used for extraterrestrial geochemical analysis, specifically for the in situ exploration of Mars on the NASA missions Mars Science Laboratory (MSL) with the ChemCam instrument [18,19] and Mars 2020 with the follow-up instrument SuperCam [20,21], and with the MarsCode instrument, which is part of the Chinese Tianwen-1 mission [22]. Even though on distant Mars LIBS measurements can take a little longer, several minutes, depending on the number of points [23], the ChemCam instrument accumulated a big and still growing dataset (more than 930,000 LIBS spectra from more than 3500 individual targets) since landing in 2012 [24]. Furthermore, with this extraterrestrial LIBS data, approaches without calibration models based on statistics have proven to be a useful support for the interpretation of the data [15,16,17,25]. With this perspective, geomaterial analysis with handheld LIBS instrumentation could also be performed by astronauts on the Moon.

For this study, we analyzed LIBS data, which were collected during an interdisciplinary summer school held on Vulcano (Eolian Islands, Italy) in June 2019. The summer school brought together different engineering and scientific techniques with the goal of developing strategies for robotic exploration of extreme environments [26]. Vulcano is the third largest and southernmost island of the Aeolian archipelago in the Tyrrhenian Sea, and the surface morphology of its central La Fossa caldera is similar to that of extremely dry and hostile planets, such as Mars and Venus, as well as the Moon. Vulcano is a remarkable training site to test instruments, rovers, or data processing techniques for planetary exploration by comparison of field and laboratory data. Furthermore, robotic missions for terrain analysis, locomotion and drone mapping, and sampling campaigns were conducted in the extreme environments of La Fossa and the coastal waters around Vulcanello, a small islet connected at the north [26].

The focus of this work is on explorative data investigation of LIBS data collected at seven different sites on Vulcano. We will not go into detail of geological interpretations but will highlight different types of targets which were identified in the analysis. To be more specific, we apply conventional PCA and compare the results with an alternative approach, the interesting features finder (IFF). This was proposed and first introduced by Wu et al. [27], where they applied the IFF to LIBS imaging data with 47,800 spectra. The main idea of this approach is to use the concept of the convex hull in order to identify pure spectra and spectra with emissions of minor and trace elements which do not contribute significantly to the variance in the dataset in contrast to the PCA approach (more details in Section 2.2). We will evaluate if and how both methods can be employed to identify unique compositions as well as trends based on chemical composition in the whole dataset.

## 2. Methods

As mentioned above, we will present the results of exploratory data analysis techniques applied to LIBS data collected in the field. Before, we will provide more details about the LIBS handheld instrumentation and the two analysis methods, PCA and IFF.

### 2.1. LIBS Handheld Device

For the LIBS measurements, a commercial handheld LIBS instrument, the Sci-Aps Z-300, was used. It consists of a Nd:YAG laser (working at its fundamental wavelength of 1064 nm, repetition rate of 10 Hz, laser energy per pulse of 5–6 mJ) and three spectrometers which cover together a spectral range of 190–950 nm. The device and other models of it are popular, in particular in the steel industry, but are used more and more also for the analysis of geological targets. A recent overview of studies using this device can be found in [6]. For our study, we used the same measurement parameters as described in [28]; one measurement refers to a 3 × 4 raster with five successive laser pulses at each point. The spectra of the plasmas induced by the first two laser pulses are discarded, known as “cleaning shots”. The emission of each plasma is recorded with a delay of 630 ns after plasma ignition and with an integration time of 1 ms. The spectra of each point are accumulated and from the whole raster, thus, from one measurement, the average spectrum is taken. In all measurements, the plasma evolved in a constant flow of Ar gas induced by the instrument in order to enhance emission line intensities [29]. Although the instrument is handy and easy to use, there are some challenges regarding the positioning of the device as reported in [28]. In the field, rock surfaces are rarely flat, which makes it impossible to measure every sample with the optimal focus position as the measurement window of the instrument is located within a flat surface which should be completely in contact with the sample. However, it was shown that qualitative differences in composition can still be identified within such a field LIBS dataset [28].

### 2.2. Data Analysis Techniques

PCA is a matrix decomposition technique, which can be used to reduce complexity in high-dimensional data [13]. PCA identifies successively axes along which the samples in the dataset have the highest variance in descending order. These axes, also called principal components (PC), are the basis of a new space with fewer dimensions to which the dataset is rotated. The rows of the rotation matrix are called loadings and the new coordinates are the scores. Applied to LIBS data, the loadings can identify correlations and anti-correlations of spectral features while the scores can reveal similar targets, i.e., clusters and patterns in the dataset. The application of PCA to LIBS data can have varying intentions reaching from preprocessing steps such as outliers filtering over dimensionality reduction to classification [14]. Usually, major compounds of a sample have the strongest contribution to the variance in a LIBS dataset, in particular when they have strong emission lines. Consequently, PCA results may be dominated by these major elements while trace compounds, which do not largely contribute to the variance, are not always detected.

As mentioned earlier, the interesting features finder (IFF) was first introduced in Wu et al. [27], and for a detailed description of the principle we would like to refer the reader to that work. Briefly, the idea is based on the principle of the convex hull with the objective to also identify spectra showing trace compounds with rather weak emission lines, which do not largely contribute to the variance. To follow the IFF idea, one has to think of LIBS spectra as objects in a high-dimensional space, to be specific with as many dimensions as the length of a spectrum. This initial space is also the one from which the LIBS spectra are rotated to PCA space, as described above. In this space, the convex hull is the surface of the object that is created if we imagine a balloon containing all the data points, i.e., the LIBS spectra, and from which the air is deflated. What is mathematically important about this is that all data points that are inside can be represented as linear combinations of data points that are part of the convex hull. With this approach, minor compounds and traces can also be expected to be part of the convex hull besides the major compounds. In practice, the challenge is to find the convex hull, and Wu et al.’s IFF approach [27] can be a way to do this. It works by creating random vectors of length of a spectrum and project all the data on it. The spectra with the most extreme coordinates are registered and this is repeated multiple times for different random vectors. The spectra, which were most often registered, are considered as being part of the convex hull. The further procedure is explained directly using our dataset in Section 5.2.

## 3. Measurement Location

**Figure 1:**
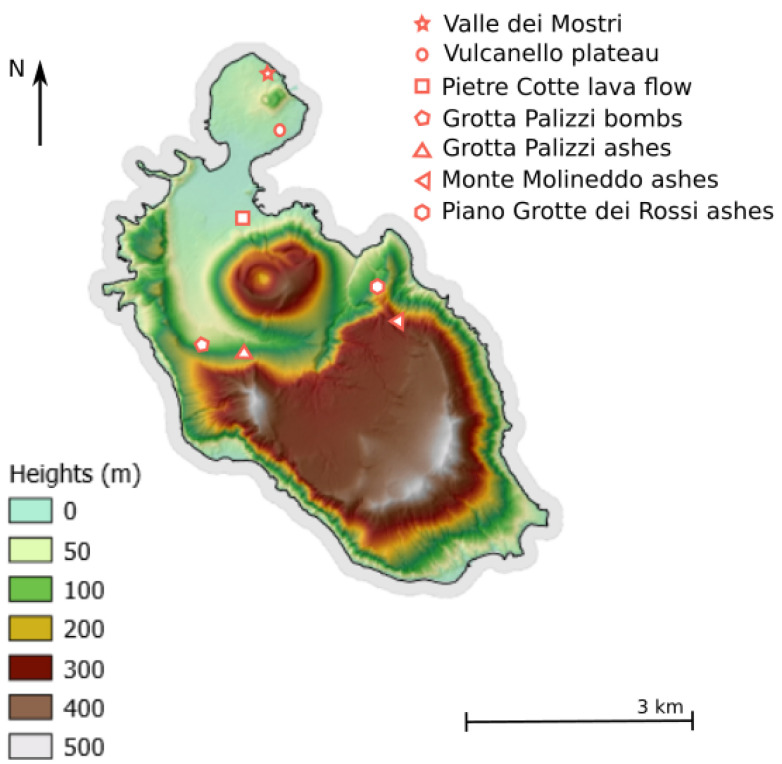
Overview of measurements sites on the island of Vulcano. The sites were chosen to be fresh, well-exposed outcrops and are described in the main text.

The island of Vulcano is located in the Aeolian archipelago, a volcanic arc in the Thyrrenian sea, just north from the island of Sicily, connected to the subduction of oceanic crust beneath the Calabrian arc [30]. Its evolution consisted in the rising of three different volcanic structures in the last 127 ka; the older structure is the Piano to the south of the island, then La Fossa caldera formed, and, finally, the Vulcanello peninsula (north of the island) was formed. On the island, a large chemical variability of magmatic products is present, from low-silica (basalts) to high silica (rhyolite) products, including alkaline products, such as shoshonite and trachyte [31]. Data for this study were collected at seven different sites shown in Figure 1. They are mainly located in the northern part of the island including the peninsula Vulcanello. With this multi-sites dataset, we ensure to cover different origins and a variety of target types as it includes LIBS data from resistant but also loose ash layers, volcanic rocks with inclusions, altered features, glassy materials, and more. Each location is described and put in context below:

- **Valle dei Mostri**: Measurements were performed on altered latite-trachite lava flows [32] belonging to the latter activity of vulcanello peninsula, more precisely identified as Vulcanello 3F in other works [33].
- **Vulcanello plateau**: Shoshonitic basement of the Vulcanello peninsula, here present as a meters-thick lava flow (Vu1b in [32]). Their composition is relatively high in alkaline content, having K_2_O + Na_2_O > 8%wt [34].
- **Pietre Cotte lava flow**: texturally heterogeneous and quasi-obsidianaceous lava flow, with a rhyolitic glassy groundmass that contains lati-trachytic enclaves [35].
- **Grotta Palizzi**: In the location known as *Vallonazzo* (rough valley) two different sets of measurements were conducted on products from the Grotta Palizzi formation (GP2a in [32]). The former set of analyses was performed on consolidated ashes organized in thinly-bedded/cross-stratified fashion labeled as **Grotta Palizzi ashes**. At the top of this formation, also pumiceous lapilli and bread-crust bombs are present and are here characterized in the second set of analyses named **Grotta Palizzi bombs**.
- **Monte Molineddo ashes**: This is a consolidated, thinly-bedded vari-colored ashes outcrop belonging to the Monte Molineddo Formation (ml in [32])
- **Piano Grotte dei Rossi ashes**: Although located close to the Monte Molineddo ashes this outcrop is different in geomorphology as it consists of dark, planar to cross-bedded ashes belonging to the Piano Grotte dei Rossi Formation (gr in [32]).

**Figure 2:**
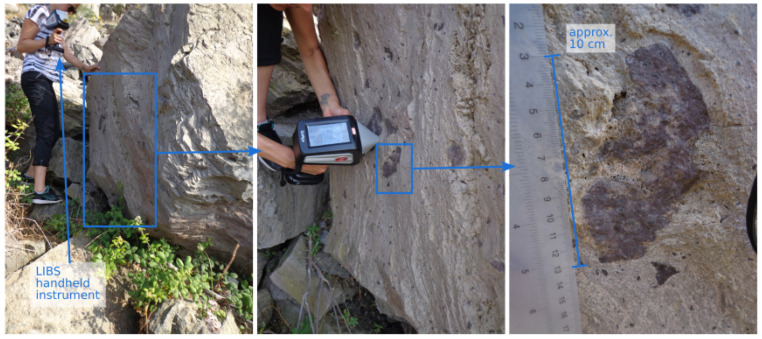
Example of in situ LIBS measurements taken on a block of the Pietre Cotte lava flow. The left image shows the context, the middle image shows how the host rock has been measured and the right picture shows a close-up of an inclusion with scaling. Several LIBS measurements were performed on this block.

As an example of a measurement site and a typical LIBS measurement, the context of a block of the Pietre Cotte lava flow is shown in Figure 2. The positioning of the hand-held LIBS instrument can also be seen, as well as a close-up view of an inclusion.

## 4. Dataset and Data Processing

In total, the dataset contains 507 LIBS spectra from the different measurement locations on the island. However, not all of them are of good quality, meaning that some have low signal-to-noise ratios and/or mainly Ar emission lines from the surrounding atmosphere. Possible reasons are discussed in [28] and can be briefly summarized as too large distances between sample and target (see Section 2.1) and sub-optimal laser-sample coupling related to the physical matrix, e.g., loose or brittle. Following [28], we automated the identification of bad quality spectra by calculating the ratio of summed intensities below and above 650 nm and also the summed intensity of the whole spectrum. Only those spectra with a ratio below 0.8 and summed total counts larger than $0.4\times 10^{6}$ were kept. As a result, 9% of the data were discarded, more details about the numbers per measurement site are summarized in Table 1. One observation to mention here is that the highest rate of discarded spectra is found for the Grotta Palizzi ashes (36% discarded data), whereas no spectrum of insufficient quality was identified for the Pietre cotte lava flow. This difference is likely an effect of different physical matrices of the samples found at the two measurement sites. While the ashes of the Grotta Palizzi site are loose and brittle which can impede the laser-sample coupling, the glass-like materials of the Pietre cotte lava flow are solid which is beneficial for the ablation process and the subsequent formation of a bright LIBS plasma [4]. A next step in the data pre-processing is the masking of Ar emission lines which was also already introduced and described in our previous work [28]. Those emission lines originate from the Ar atmosphere and do not contain relevant information about the sample’s composition, but can strongly influence statistical data exploration methods. To avoid this, we masked all relevant Ar emission lines. In the last step before the actual data analysis, the spectra are standardized so that each spectrum has a mean value of zero and a standard deviation of one.

## 5. Results

### 5.1. PCA Results

**Figure 3:**
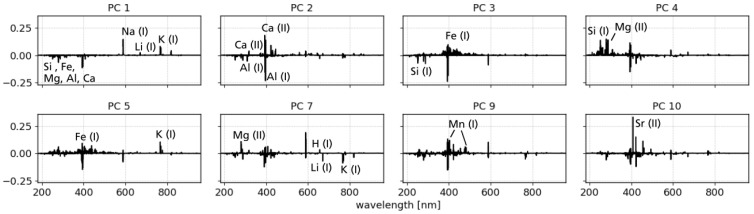
Loadings of the PCA components indicating correlations among emission lines of elements. Even though the emission lines cannot be seen in detail, we have annotated those that seem to be characteristic of the corresponding component. Not shown are the loadings of PC 6 and PC 8 as they do not contain relevant information for this study.

The loadings of the PCA for selected components are shown in Figure 3. They indicate positive and negative correlations of pixels, i.e., wavelengths, and can assist interpretations regarding composition. Details are not discussed here, but in Section 5.3 where IFF and PCA results are evaluated together. Nevertheless, we will mention a few parameters and observations of the PCA at this point. The summed explained variance, which increases with the number of components, is often used as an indicator of how many components are needed for a model to best describe the data without over- or underfitting [14]. For our dataset, it takes 16 components to explain 95% of the variance. Nevertheless, we mainly investigated the first 10 components in more detail, since it becomes apparent when looking at the loadings that not only varying line strengths are responsible for the variance in the dataset. Another factor contributing to the variance is varying line positions, which may be due to different wavelength calibrations or different plasma conditions, such as different electron densities. Since we are mainly interested in variations in the elemental composition of the targets, as evidenced by varying line strengths, we will not elaborate on components that show influences of line shifts.

### 5.2. IFF Results

**Figure 4:**
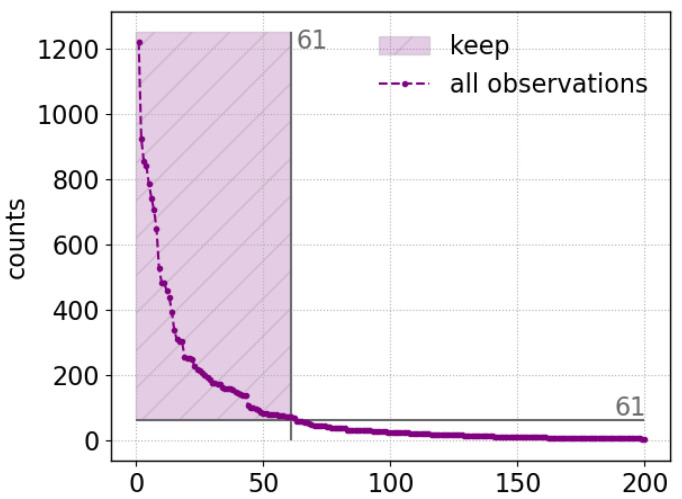
IFF selection frequency as counts for the 200 spectra which were selected most often in decreasing order. All observations selected 61 times or more often (dashed purple area) were kept and investigated further. This resulted in 61 selected spectra (it is coincidence that both numbers are 61).

The IFF analysis was applied to the same dataset as the PCA with the same data pre-processings as described in Section 4. As already mentioned in Section 2.2, we will follow the approach introduced in [27]. One LIBS spectrum is a one-dimensional vector of length 23,431 and the standardized intensity values range between −3 and 30. Consequently, 10,000 one-dimensional vectors of same length and with random entries of values between −3 and 30 were created. All spectra were projected on each of those vectors and it was recorded which spectra have the most extreme, thus minimum and maximum, coordinates on them. Each time a spectrum has one of the most extreme projection lengths, its selection frequency is updated. In the end, the highest reached selection frequency of one spectrum was 1221. As a threshold for a spectrum being identified as *interesting* by the IFF, we took 5% of this number which corresponds to a selection frequency of 61. Therefore, observations selected 61 or more often were kept. With this threshold, we end up with 61 spectra (coincidence that both numbers are 61) which will be noted as IFF spectra (IFFs) in the following. Figure 4 shows the selection frequencies of the 200 most selected spectra in decreasing order.

**Figure 5:**
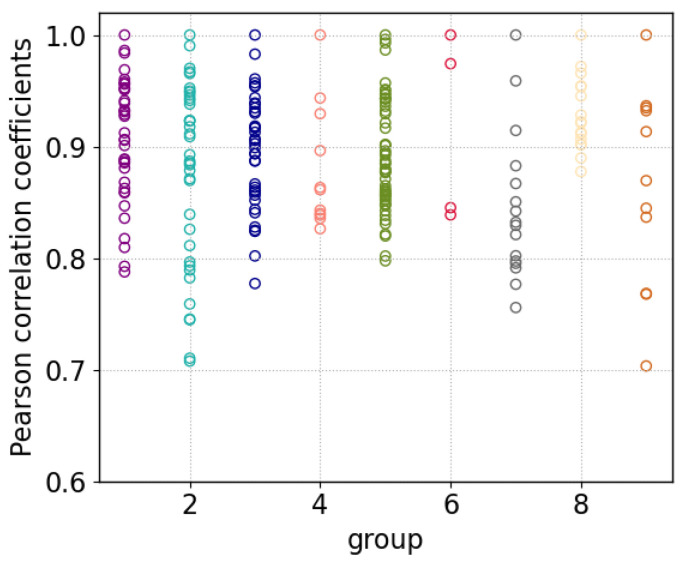
Pearson correlation coefficients shown for each subgroup of the group of 61 spectra identified by the IFF. The coefficients give pairwise correlations of all possible combinations in the subgroups. Almost all correlations are larger than 0.75. The different colors correspond to the subgroups.

**Figure 6:**
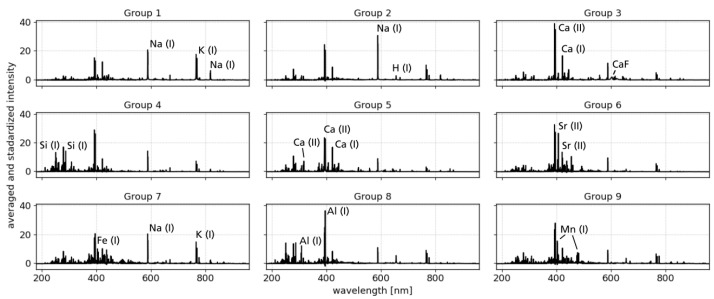
Mean spectrum for each IFF subgroup identified by a combination of PCA and hierarchical clustering. The emissions that characterize the group and distinguish it from others were marked in the spectra.

It is possible that this selection of 61 IFFs contains groups of similar spectra [27]. Thus, to investigate correlations, we consult a PCA analysis followed by hierarchical clustering. Without going into too much detail, it can be said about the PCA that with 10 components more than 90% of the variance is explained. Hierarchical cluster analysis was thus applied to the scores of the first 10 components. Type ward was used as linkage and, with a cut-off factor of 0.3, a total of 9 groups among the IFFs could be identified. Within the groups, Pearson correlations coefficients among all members were computed and can be seen in Figure 5. Almost all correlations are larger than 0.75, indicating in-group spectral similarity. Even though the spectra of a group also show smaller deviations, the high correlations legitimize the fact that mean spectra are discussed in the following. The mean spectra of each IFF group are shown in Figure 6.

### 5.3. Combined Results

**Figure 7:**
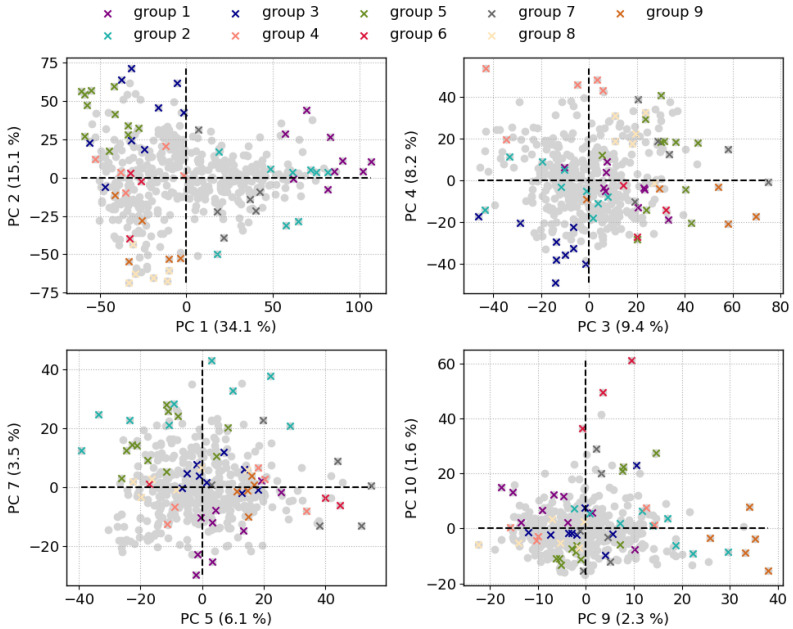
PCA score plots of all observations in the dataset. The number in brackets at the axes labels gives the explained variance by the PC. The colored crosses mark spectra which are members of the IFF groups. We focus here on PCs which more or less separate IFF groups, as a result, scores on PC 6 and PC 8 are not shown.

Finally, the results of the PCA and IFF are discussed together in the following, which will be mainly based on the loadings of the PCA in Figure 3 and the mean spectra of the IFF groups in Figure 6. Additionally, Figure 7 shows the PCA scores of selected PC components where the members of the IFF groups are highlighted. In the PC 1 vs. PC 2 plot, it is noticeable that the IFF spectra are rather in the outer PCA space and almost encircle the other observations. This may already indicate that the IFF can identify extreme observations. The combined results are presented in the manner of a listing based on the PCA components. We restrict the discussion to selected observations that provide a good basis for comparing the two methods.

- **PC 1** has positive correlations with emission lines from alkali elements, such as Li, Na, and K. The members of the IFF groups 1 and 2 and to a lesser extent also group 7 have mainly positive scores on PC 1 which is in agreement with their mean spectra showing strong emission lines of these elements.
- **PC 2** is dominated by positive correlations with Ca emission lines and negative correlations with Al lines. In PCA space, PC 2 separates the IFF groups 3 and 5 from groups 8 and 9. In particular, the mean spectrum of group 8 shows strong lines of aluminum, whereas the mean spectra of group 3 and 5 show distinct lines of Ca.
- **PC 4** has the strongest positive correlation with Si lines but also with Li and Mg. Compared to other components, PC 4 has a strong anti-correlation with the molecular emission band of CaF. Regarding the IFF groups, PC 4 splits groups 3 and 4 apart, which is consistent when looking at the mean spectra and the already discussed correlations.
- **PC 5** is positively correlated with Fe emission lines, mainly neutral ones, and the K emission lines. Regarding the scores on PC 5, the main observation is that group 7 spectra have solely positive values and also the largest ones. This agrees with Fe emission lines in the mean spectrum of group 7. However, the mean spectrum has similar strong Na emission as K emission, although they are anti-correlated on PC 5.
- **PC 7** separates the IFF groups 1 and 2 which are both dominated by emission lines of alkali elements. What distinguishes the two is evident on PC 7: emissions from Li and K are anti-correlated whereas those from Na have a positive correlation with PC 7. The scores of group 1 are negative and indeed the mean spectrum of group 1 also shows stronger lines of K than that of group 2.
- **PC 9** is notable for positive correlations with Mn emission lines. This is in excellent agreement with the mean spectrum of group 9, whose members all have positive scores on PC 9.
- **PC 10** also shows clear correlations here with emission lines of Sr. This is matched by the members of group 6, who all show strong Sr lines and at the same time have positive score values on PC 10.

In general, the combined results show that both methods can identify single extreme spectra but also transitions between different types of concentrations. Examples for the first kind of spectra are members of the IFF groups 6 and 9; the special features of these spectra are strong lines of Sr and Mn, respectively. In PCA space, exactly these spectra have high scores on PC 9 and 10 showing Sr and Mn lines in their loadings while the rest of the spectra have only small to moderate score values. The other type of observation are IFF group spectra, which could represent end-members as they are located at outer points in PCA space. Other spectra align between those observations, which indicates the existence of compositional transitions between end-members. Examples are IFF groups 1, 2, 3, 5, and 8, see PC 1 vs. PC 2 plot in Figure 7.

## 6. Discussion

In contrast to the study by Wu et al. [27], we could not identify a spectrum with the IFF with emissions from minor or trace elements which were not already revealed in the first 10 PCA loadings of our data. This is not surprising, however, since the LIBS data investigated in the work by Wu et al. [27] differ greatly from our LIBS field data. They worked with LIBS imaging data where significantly more spectra were measured in a laboratory setup with the experimental conditions being well controlled and nominally constant. The sample matrix was the same for each measurement point and any spectral variation indicates a change in composition. Our field dataset, on the other hand, is much smaller and multiple factors besides changing composition can be responsible for spectral variations. For our use case, neither PCA nor IFF was clearly a superior analysis approach in comparison to the other. Furthermore, the combination of the two methods is found most useful in exploring the data. The IFF provides complementary information to the PCA; it enables the identification of endmembers and supports the interpretation of correlations.

In terms of geology and conclusions that can be drawn from the results, no distinct difference in chemical composition characteristic of any of the measurement sites can be observed. Nevertheless, we want to briefly mention a few observations, even if the focus of this study is on the data analysis methods. The first PC, which explains the most variance in the dataset (34.1%), separates spectra with strong emission lines of alkali metals (K, Li, Na) from those which have stronger lines of other major rock forming elements (Si, Fe, Mg, Al, Ca), see loadings of PC 1 in Figure 3. Not all, but several spectra measured at the Vulcanello plateau site have high scores on PC 1 which is in line with the high alkaline content of this lava flow [34]. That much of the variance in the dataset can be linked to alkaline emission lines is in agreement with the variable alkaline content of ashes and rocks on Vulcano as reported in [32].

**Figure 8:**
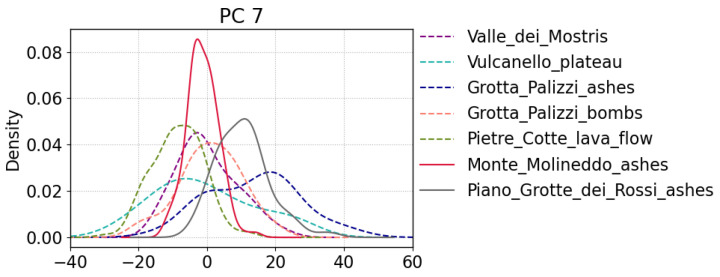
Density curves of PC 7 score values for each measurement site. For better visualization the curves of the Monte Molineddo and the Piano Grotte dei Rossi ashes are shown as solid lines. The curves were generated with Gaussian kernels and the bandwidths were selected following Scott’s rule. Note that the area below each curve is normalized to one.

Another observation relates to PC 7 and, although it explains only 3.5% of the variance in the dataset, we want to use it for a more detailed discussion. As described above, PC 7 can distinguish between K- and Na-rich spectra, where K emission lines are negatively correlated and Na lines are positively correlated with PC 7 (see Figure 3). The distributions of the PC 7 score values for each measurement site are shown as density curves in Figure 8. The solid lines correspond to the Piano Grotte dei Rossi ashes (gray line) and the Monte Molineddo ashes (red line) outcrop. Spectra of the first have mostly positive score values on PC 7 while those of the latter have small but variable score values around zero. This means that the Piano Grotte dei Rossi spectra have less K in comparison to Monte Molineddo ashes. This is in agreement with the chemistry of Piano Grotte dei Rossi formation being latitic to rhyolitic while that of the Monte Molineddo formation is shoshonitic to trachytic [32]. As mentioned above, no significant differences could be found in the PCA and IFF results between the measurements sites. Nevertheless, for the sake of completeness, it should be mentioned that the density plots of the other PCs (not shown), when the data are grouped by site, also show weak trends in some cases. For example, spectra from the Monte Molineddo ashes have predominantly negative scores on PC 1. Furthermore, the observations from the Vulcanello plateau have overall the highest positive scores on P 2 and PC 3, while Monte Molineddo ashes and Piano Grotte dei Rossi ashes have the largest negative scores on PC 2, indicating a possible difference in Ca abundances between these sites. The Valle dei Mostri measurements have slightly higher positive score values on PC 5. We would like to emphasize that these trends in the density plots are weak and, therefore, do not apply to all the measured targets for the sites.

It turns out that there are three IFF groups, namely groups 6, 8, and 9 which were only observed at one of the locations, the Monte Molineddo ashes outcrop. While group 6 (Sr-rich) and group 9 (Mn-rich) represent somehow unique compositions, group 8 (Al-rich) can be interpreted as an end-member spanning a compositional trend.

## 7. Summary and Conclusions

After data cleaning based on [28] and dedicated data preprocessing by the removal of the Ar emission lines, two explorative data analysis methods, namely PCA and the IFF, were applied to LIBS data measured with handheld instrumentation at seven different sites on the island Vulcano, Italy. The dataset covers a variety of different target types that vary in both their origin and physical matrix. With the IFF [27], no minor or trace elements could be identified beyond those seen with PCA; however, the methods complement each other well and their combination benefits unsupervised data exploration. The dataset was found to differ mostly in alkali content, namely Na, K, and Li, as well as Al, Ca, and Mg. Furthermore, Sr and Mn were detected in several spectra of the dataset.

Like in our previous study [28], some kind of lessons-learned can be derived from the analysis of the LIBS dataset collected in 2019 on Vulcano. For future field campaigns using the handheld LIBS device, we recommend the measurement of more points per unique target and to pay extra attention that the measuring head of the handheld is placed well in contact with the sample.

## Figures and Tables

**Table 1:** Overview of measurement sites on Vulcano and the number of measured spectra at each location before and after the data quality check. The number (#) of spectra measured at each site depended on the accessibility and availability of interesting targets, and, therefore, differs between sites.

Site	# of LIBS Spectra Measured	# of LIBS Spectra after Outsorting
Valle dei Mostri	39	32
Vulcanello plateau	32	28
Pietre cotte lava flow	123	123
Grotta Palizzi bombs	77	71
Grotta Palizzi ashes	64	41
Monte Molineddo ashes	116	113
Piano Grotte dei Rossi ashes	56	55
Total	507	463

## Data Availability

Data can be made available upon request.

## Abbreviations

The following abbreviations are used in this manuscript:

LIBS	Laser-induced breakdown spectroscopy
MSL	Mars Science Laboratory
IFF	Interesting features finder
IFFs	Interesting features finder spectra
PCA	Principal component analysis
PC	Principal component
